# Regulation of proliferation and invasion by the IGF signalling pathway in Epstein‐Barr virus‐positive gastric cancer

**DOI:** 10.1111/jcmm.13859

**Published:** 2018-09-24

**Authors:** Inhye Jeong, Sun Kyoung Kang, Woo Sun Kwon, Hyun Jeong Kim, Kyoo Hyun Kim, Hyun Myong Kim, Andre Lee, Suk Kyeong Lee, Thomas Bogenrieder, Hyun Cheol Chung, Sun Young Rha

**Affiliations:** ^1^ Brain Korea 21 PLUS Project for Medical Science Yonsei University College of Medicine Seoul Korea; ^2^ Songdang Institute for Cancer Research Yonsei University College of Medicine Seoul Korea; ^3^ Yonsei University College of Medicine Seoul Korea; ^4^ Department of Biological Sciences Columbia University New York New York; ^5^ Department of Medical Lifescience College of Medicine The Catholic University of Korea Seoul Korea; ^6^ Boehringer Ingelheim RCV GmbH & Co KG Vienna Austria; ^7^ Department of Urology University Hospital Grosshadern Ludwig‐Maximilians‐University Munich Germany; ^8^ Department of Internal Medicine Yonsei Cancer Center Yonsei University College of Medicine Seoul Korea

**Keywords:** BI836845, epstein‐barr virus, gastric cancer, IGF signalling pathway

## Abstract

Several carcinomas including gastric cancer have been reported to contain Epstein‐Barr virus (EBV) infection. EBV‐associated gastric cancer (EBVaGC) is classified as one of four molecular subtypes of gastric cancer by The Cancer Genome Atlas (TCGA) group with increased immune‐related signatures. Identification of EBV‐dependent pathways with significant biological roles is needed for EBVaGC. To compare the biological changes between AGS gastric epithelial cells and EBV‐infected AGS (AGS‐EBV) cells, proliferation assay, CCK‐8 assay, invasion assay, cell cycle analysis, RT‐PCR, Western blot and ELISA were performed. BI836845, a humanized insulin‐like growth factor (IGF) ligand‐neutralizing antibody, was used for IGF‐related signalling pathway inhibition. AGS‐EBV cells showed slower proliferating rate and higher sensitivity to BI836845 compared to AGS cells. Moreover, invasiveness of AGS‐EBV was increased than that of AGS, and BI836845 treatment significantly decreased the invasiveness of AGS‐EBV. Although no apoptosis was detected, entry into the S phase of the cell cycle was delayed in BI836845‐treated AGS‐EBV cells. In conclusion, AGS‐EBV cells seem to modulate their proliferation and invasion through the IGF signalling pathway. Inhibition of the IGF signalling pathway therefore could be a potential therapeutic strategy for EBVaGC.

## 1 INTRODUCTION

Gastric cancer is presented as the third highest cumulative risk of cancers worldwide in 2012.^1^ To improve our understanding of gastric cancer and to provide a roadmap for clinical trials of targeted therapy, The Cancer Genome Atlas (TCGA) group classified gastric carcinoma into four molecular subtypes: Epstein‐Barr virus (EBV)‐positive, microsatellite instability (MSI), genomically stable (GS) and chromosomal instability (CIN).^2^

EBV, the focus of this study, was first detected in a Burkitt's lymphoma cell line. Since then, many studies have found EBV to be associated with several well‐known human malignancies such as nasopharyngeal carcinoma (NPC), Hodgkin's lymphoma and Epstein‐Barr virus‐associated gastric cancer (EBVaGC).^3^, ^4^ Incidence of EBVaGC is reported to be approximately 10% of globally detected gastric cancers. In addition, clinicopathological characteristics such as undifferentiated type^5^ and CpG island hypermethylation^6^ are reported to be high in EBVaGC. Prognosis of EBVaGC is also better than that of EBV‐negative cases because lymph node metastasis in EBVaGC is significantly less frequent than in EBV‐negative gastric cancer.^7^

Many studies characterizing EBVaGC have been reported since the 1990s.^8^ Most studies have focused on finding EBV‐specific genes and their biological functions,^9^, ^10^ related microRNAs^11^, ^12^ and chemo‐resistance mechanisms.^13^, ^14^ Recently, high‐throughput assays were attempted to uncover regulatory mechanisms in EBVaGC.^15^ However, only a few studies on target‐specific pathways in EBVaGC have been reported. One report has suggested that sequential combination is needed to improve the sensitivity of 5‐fluororacil, one of adjuvant therapy agents in solid tumour, sensitivity when using PI3K inhibitors in EBV‐positive gastric cancer cell lines.^16^

Several studies have suggested that the insulin‐like growth factor‐1 receptor (IGF‐1R) pathway is an essential target of EBV‐positive cancer such as NPC.^17^ IGF‐related ligands (IGF‐1, IGF‐2 and insulin) and insulin‐like growth factor binding proteins (IGFBPs) are produced the liver that are stimulated by growth hormone (GH). Mainly, a complex of circulating IGFBPs and acid‐labile subunit (ALS) prolongs the half‐life of IGF‐related ligands and delivers them to a complete receptor. Activated IGF‐1R by IGF‐1 and IGF‐2 plays a critical role in proliferation, migration and invasion. Many cancers express IGF‐1R and 75.2% of stomach cancer tissue expresses IGF‐1R, which may be the cause of poor prognosis.^18^

Several IGF‐1R‐targeted drugs were developed based on the importance of the IGF‐1R signalling pathway, but few proved to be effective. Considerable biological restrictions of IGF‐1R are the reasons why development of IGF‐1R pathway‐targeting drugs has been difficult.^19^ The major problem of targeting IGF‐1R lies in its sequence similarity to the insulin receptor, which may lead to metabolic dysfunction. Mainly, insulin receptor isoform A (IR‐A) activated by IGF‐2 is known to promote oncogenic signalling. To overcome blocking that action without interfering the insulin axis insulin receptor activation, BI836845 (Xentuzumab), a drug that targets both IGF‐1 and IGF‐2, was developed and there are many ongoing clinical trials on various type of tumour.^20^

In this study, we investigated the IGF‐1R pathway and related biological roles in EBVaGC using BI836845. We also compared biological changes after IGF‐1R signalling pathway inhibition in AGS and AGS‐EBV cell lines.

## 2 METHODS

### 2.1 Materials

BI836845, a humanized IGF ligand‐neutralizing antibody, was kindly provided by Dr. Ulrike Weyer‐Czernilofsky (Boehringer Ingelheim). Anti‐pIGF‐1R, anti‐IGF‐1R, anti‐pAkt‐Thr308, anti‐pAkt‐Ser473, anti‐pERK and anti‐Snail antibodies were purchased from Cell Signaling (Danvers). Anti‐IGFBP‐3, anti‐IGFBP‐6, anti‐ERK, anti‐vimentin (Santa Cruz Biotechnology, Dallas), anti‐E‐cadherin (BD biosciences, Franklin Lakes) and anti‐α‐tubulin (Sigma‐Aldrich, Saint Louis) antibodies were also purchased.

### 2.2 Cell lines

Human gastric epithelial cancer cell line AGS (ATCC No. CRL‐1739) was obtained from the American Type Culture Collection. AGS‐EBV cell line was infected with green fluorescent protein (GFP)‐tagged EBV made from Akata cells^21^ (Supplementary 1A and B). AGS cells were maintained in EMEM supplemented with heat‐inactivated 10% foetal bovine serum containing 100 Units/mL penicillin and 100 μg/mL streptomycin. AGS‐EBV cells were cultured in the same culture medium with the addition of 400 μg/mL G418 (A.G. Scientific, San Diego). Cells were incubated at 37°C in a humidified atmosphere of 5% CO_2_.

### 2.3 Proliferation assay

For proliferation assays, 2.0 × 10^3^ cells were seeded in 24‐well plates. The wells were filled with fresh media and 10 μg/mL BI836845 the day after seeding and repeated every 3 days. Samples from triplicate wells were harvested every day, and cells were counted after Trypan blue staining. Growth curves were plotted as cell numbers versus time.

### 2.4 Cell viability

Cell viability was determined using a Cell Counting Kit‐8 (Dojindo, Kumamoto, Japan) according to the manufacturer's instructions. Briefly, 5.0 × 10^4^ cells were seeded in 96‐well plates and incubated for 24 hours. Serial diluent of BI836845 (0, 0.01, 0.1, 1, 10, 100 μg/mL) was treated to each well for 72 hours. Next, 10 μL of CCK‐8 solution was added to each well and incubated for 2 hours at 37°C. Absorbance was determined at 450 nm using microplate reader (TECAN, Männedorf, Switzerland).

### 2.5 Invasion assay

Trans‐well invasion chambers were pre‐coated with 500 ng/mL Matrigel (Corning, Corning, USA) for 6 hours. The medium at the bottom of the 24‐well plate was replaced with fresh medium containing 10% FBS, and 2 × 10^4^ cells in serum free media were added to the top chamber. After incubation for 24 hours at 37°C, the cells were then fixed with 4% formaldehyde for 2 minutes. The chambers were then washed with PBS and stained with 1% crystal violet for 10 minutes. After cleaning and drying the chamber membrane, cells were counted using an inverted microscope (Zeiss, Oberkochen, Germany).

### 2.6 RT‐PCR

Total RNA was isolated using Trizol (Thermo Fisher Scientific, Waltham) according to the manufacturer's instructions. Total RNA (2 μg) was used for reverse transcription with Superscript II reagent kit (Invitrogen, Carlsbad). IGF‐1R, IGF‐1, IGF‐2, IGFBP‐3, IGFBP‐6 and glyceraldehyde‐3‐phosphate dehydrogenase (GAPDH) primers were designed as follows: IGF‐1R forward, 5′‐TACAACTACGCCCTGGTCATC‐3′, and reverse, 5′‐CTTCTCACACATCGGCTTCTC‐3′; IGF‐1 forward, 5′‐CTGGAGATGTATTGCGCACC‐3′, and IGF‐1 reverse, 5′‐ CTTGTTGGTAGATGGGGGCTG‐3′; IGF‐2 forward, 5′‐ TCCCCTGATTGCTCTACCCA ‐3′, and IGF‐2 reverse, 5′‐TTCCGATTGCTGGCCATCTC‐3′; IGFBP‐3 forward, 5′‐AAGACAGCCAGCGCTACAAAG‐3′, and IGFBP‐3 reverse, 5′‐ TACGGCAGGGACCATATTCTG‐3′; IGFBP‐6 forward, 5′‐ ATGCCGTAGACATCTGGACTCA‐3′, and IGFBP‐6 reverse, 5′‐ AGAAGCCTCGATGGTCACAATT‐3′; and GAPDH forward, 5′‐CCATGGAGAAGGCTGGGG‐3′, and reverse, 5′‐CAAAGTTGTCATGGATGACC‐3′. PCR was performed on a thermal cycler (Eppendorf, Hamburg, Germany) using the appropriate annealing temperature. PCR products were examined using agarose gel electrophoresis and exposed to a UV detector to confirm the presence of a single amplification product.

### 2.7 ELISA

Concentration of IGF‐1 and IGF‐2 was measured using human ELISA kit (Abnova, Taipei City, Taiwan) according to the manufacturer′s instructions. For the sample preparation, 2.5 μg of total lysate protein and 5 μg of total secreted protein were quantified by BCA assay. Finally, concentration of IGF‐1 and IGF‐2 was calculated at 450 nm using a microplate reader.

### 2.8 Western blotting

For Western blotting, control and BI836845‐treated cells were collected 24 hours after treatment. A total of 30 μg of whole cell protein extracts in RIPA lysis buffer were size‐fractionated using 10% SDS polyacrylamide gel electrophoresis and transferred to a polyvinylidene difluoride membrane (Bio‐Rad, Hercules). Membranes were blocked with 5% non‐fat dry milk/TBS‐T for 1 hour at room temperature and then incubated overnight with primary antibodies at 4°C. Membranes were washed five times with TBS‐T and incubated with horseradish peroxidase‐conjugated secondary antibody for 1 hour at room temperature. Blots were washed five times again, and protein signals were enhanced and detected using a chemiluminescence detection kit (Santa Cruz, Dallas). The intensity of the bands was normalized using α‐tubulin with ImageJ‐based quantification.

### 2.9 Flow cytometry

For apoptosis analysis, 5 × 10^5^ cells were seeded in 6‐well plates and incubated for 24 hours at 37°C in an incubator. BI836845 was directly added to the wells, and cells were incubated for 24 hours and 48 hours and collected in 15 mL tubes. After PBS washing, cells were double‐stained with recombinant Pacific blue‐conjugated Annexin V and propidium iodide (PI). Cells were gently vortexed and incubated for 15 minutes at room temperature in the dark. Flow cytometry analysis was performed using a FACS LSRII (BD biosciences, Franklin Lakes) with CellQuest software. Ten thousand single cells were gated and analysed for apoptosis.

Cell cycle analysis was performed in two different conditions. In the first condition, ordinary cell cycle analysis was performed. Cells were seeded in 60‐mm^2^ dishes and incubated in media with or without BI836845 after 24 hours. After further incubation for 24 and 48 hours, cells were harvested. In the second condition, cells were synchronized in G0/G1 phase after culturing in confluent monolayers under serum starvation during 48 hours. After starvation, wells were incubated with 10% FBS‐containing media, and 10 μg/mL BI836845 was added to each well. Cells harvested immediately after the 48 hours of serum starvation were labelled 0 hours; additional aliquots were harvested at the indicated time‐points. Cells harvested at either the first or the second condition were fixed in cold 70% ethanol for more than 24 hours and stained with PI (BD biosciences, Franklin Lakes. DNA contents were determined using FACS LSRII (BD biosciences), and gated 20,000 events/sample were collected for cell cycle analysis.

### 2.10 Statistical analysis

Continuous data were analysed using the Student *t* test. Differences were considered statistically significant when *P* < 0.05. The significance of dose‐ or time‐dependent change was calculated by two‐way ANOVA with the use of IBM SPSS statistics.

## 3 RESULTS

### 3.1 Expression of IGF‐related genes and proteins in EBVaGC

**Figure 1:**
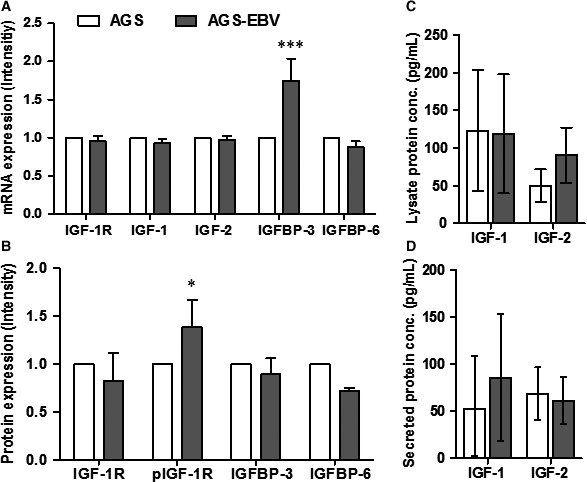
mRNA and protein expression of IGF‐related factors on AGS and AGS‐EBV. (A) mRNA expression was measured by RT‐PCR. All factors were normalized by GAPDH and divided by AGS expression level for relative quantification with SD (B) Quantification of protein expression was evaluated by Western blot. All factors were normalized by α‐tubulin, and AGS cell line was used as a control. The factors were represented as mean ± S.D (C) IGF‐1 and IGF‐2 in 2.5 μg of total lysate protein was measured by ELISA. (D) Secreted IGF ligands were quantified in 5 μg of total protein. IGF‐1 and IGF‐2 expressed with mean with 95% confidence interval. Statistical significance is represented in relation to control: AGS versus AGS‐EBV; *P < 0.05, ***P < 0.001

To evaluate IGF‐related gene and protein expression, the baseline expression levels in AGS and AGS‐EBV cells were first determined. Comparing AGS and AGS‐EBV cells, no significant differences in the mRNA levels of IGF‐1R, IGF‐1, IGF‐2 and IGFBP‐6 were observed. Interestingly, IGFBP‐3 mRNA levels in AGS‐EBV cells were 74.6 ± 28.8%, which was higher than those in AGS cells (Figure 1A).

Western blot analysis showed that, although total IGF‐1R protein levels in AGS‐EBV were 17.4 ± 28.8% lower than those in AGS cells, phospho‐IGF‐1R levels were 38.9 ± 28.1% higher than those in AGS cells (Figure 1B). In addition, IGFBP‐3 and IGFBP‐6 levels in AGS‐EBV were 10.6 ± 16.9% and 27.9 ± 3.0% lower, respectively, than in AGS cells.

To compare the expression levels of ligands, lysate and secreted IGF‐1 or IGF‐2 were measured using ELISA (Figure 1C and D). In AGS and AGS‐EBV, lysate IGF‐1 levels were similar, while lysate IGF‐2 levels were 49.9 (95% confidence interval [CI]: 28.4‐71.4) and 90.3 (95% CI: 53.7‐126.9) pg/mL, respectively (Figure 1C). In contrast, secreted IGF‐1 levels increased when EBV was present, from 52.3 to 85.4 pg/mL. Secreted IGF‐2 levels in AGS and AGS‐EBV cells were similar (Figure 1D). While no significant differences were observed between AGS and AGS‐EBV, AGS‐EBV generally showed higher expression of lysate IGF‐2 and secreted IGF‐1.

The results show that IGFBP‐3, secreted IGF‐1 and lysate IGF‐2 expression levels were increased, and phospho‐IGF‐1R was more activated, in AGS‐EBV cells. With the exception of the aforementioned factors, the expression levels of most IGF‐related factors were decreased in AGS‐EBV.

### 3.2 Effect of BI836845 on proliferation, sensitivity and invasion of AGS and AGS‐EBV cells

**Figure 2:**
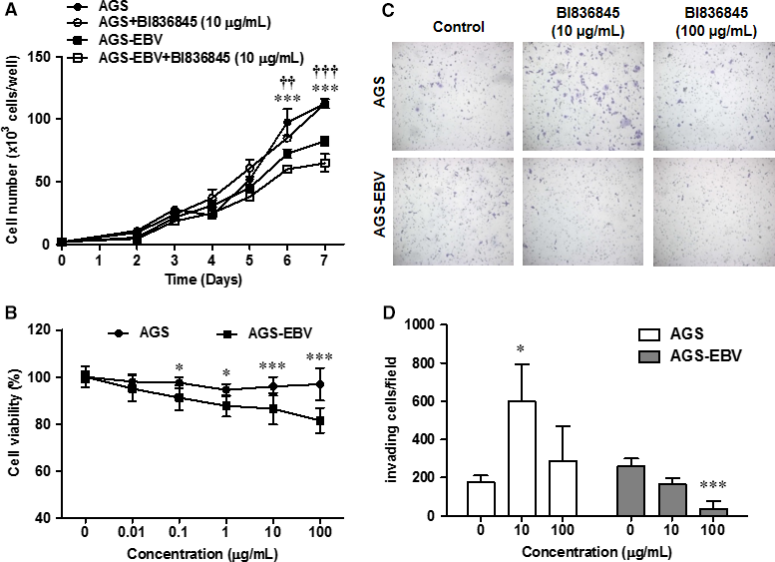
Phenotypic changes in AGS and AGS‐EBV cells after treatment with BI836845. (A) Proliferation of AGS and AGS‐EBV cells was determined with Trypan Blue exclusion assays for 7 days. (B) BI836845 sensitivity was measured using CCK‐8 assays after 72 h. (C) Representative crystal violet staining images of AGS and AGS‐EBV cells. (D) Invasive cells were counted in eight fields of three different wells. Results were normalized to control and are shown as mean ± SD Statistical significance is represented in relation to control: AGS versus AGS‐EBV, *P < 0.05, ***P < 0.001; AGS‐EBV versus AGS‐EBV treatment, ^††^ *P* < 0.01, ^†††^ *P* < 0.001

To evaluate the effect of EBV infection on AGS cells, proliferation assay was first performed. On days 6 and 7, proliferating AGS cells were at significantly higher number than AGS‐EBV cells (*P* < 0.01 and *P* < 0.001 at days 6 and 7, respectively; Figure 2A). We then evaluated the effect of BI836845 on proliferation. Interestingly, no significant difference was observed in AGS cell proliferation between the control and BI836845‐treated groups. In contrast, proliferation of AGS‐EBV cells was significantly inhibited by 10 μg/mL BI836845 treatment (Figure 2A). Also, cell viability of AGS and AGS‐EBV cells in the presence of BI836845 was evaluated 72 hours post‐treatment. The results show that AGS cells were not sensitive to the BI836845 treatment, whereas AGS‐EBV cells exhibited significant dose‐dependent inhibition (Figure 2B).

We also performed in vitro invasion assay using trans‐well chambers to determine whether EBV infection is associated with invasiveness of gastric cancer cells. The results show that the number of AGS and AGS‐EBV cells that had invaded through the membrane of the trans‐well chamber was 175.0 ± 36.0 and 259.0 ± 40.6 cells/well, respectively (*P* = 0.055), indicating that AGS‐EBV increased their baseline invasiveness compared to AGS cells. We then determined the effect of BI836845 treatment on invasiveness of both AGS and AGS‐EBV cells. AGS cells in the treatment group showed increased invasiveness compared to the AGS control group, particularly at 10 μg/mL BI836845 (*P* < 0.05). In contrast, AGS‐EBV cells presented reduced invasiveness in a dose‐dependent manner, with a significant reduction at 100 μg/mL BI836845 (*P* < 0.001) (Figure 2C,D).

In summary, EBV infection inhibited cell proliferation and cells became sensitive to BI836845. Moreover, EBV infection led to increased invasiveness which in turn was suppressed by BI836845 treatment.

### 3.3 Effect of BI836845 on mRNA and protein expression of IGF‐related factors and epithelial‐mesenchymal transition (EMT) markers

**Figure 3:**
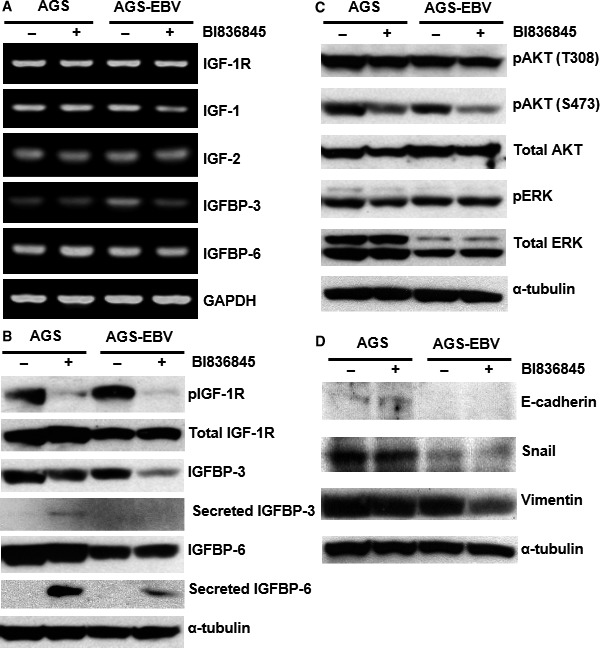
Expression of IGF‐related genes and proteins was determined using RT‐PCR and Western blotting, respectively. (A) mRNA expression levels of IGF‐relates genes were detected and normalized with GAPDH. (B) Protein expression levels of IGF‐related factors were measured. (C) Expression levels of downstream factors of the IGF‐1R signalling pathway were determined. (D) Protein expression levels of Snail and Vimentin, representative epithelial–mesenchymal transition markers, were measured using Western blotting. ‐, Control without BI836845 treatment; +, treatment with 10 μg/mL BI836845 for 24 h

We determined the effect of BI836845 on IGF‐ and EMT‐related protein and gene expression levels in AGS and AGS‐EBV cells. In BI836845‐treated AGS cells, mRNA expression levels of IGFBP‐6 were increased, whereas no change was observed in the expression levels of all the other factors. In contrast, in BI836845‐treated AGS‐EBV cells, a decrease in mRNA expression levels of IGF‐1, IGFBP‐3 and IGFBP‐6 was observed (Figure 3A).

As can be seen in Figure 3B, phospho‐IGF‐1R levels were markedly reduced after BI836845 treatment in both AGS and AGS‐EBV cells. Lysate IGFBP‐3 levels in AGS‐EBV cells also decreased after BI836845 treatment. Comparison between AGS and AGS‐EBV cells showed that IGFBP‐3 was secreted only in AGS cells. In addition, no changes in expression patterns were observed in the other factors after BI836845 treatment.

Even though total Akt levels were not changed after EBV infection, total ERK levels decreased (Figure 3C). No changes in phospho‐Akt (T308) or phospho‐ERK levels were observed between AGS and AGS‐EBV cells after BI836845 treatment. However, phospho‐Akt (S473) levels were generally reduced post‐treatment. In addition, phospho‐Akt (S473) levels in the AGS‐EBV treatment group decreased to a higher extent than those in the AGS treatment group (Figure 3C).

These results indicate that blocking IGF‐1 and IGF‐2 with BI836845 leads to inhibition of the IGF‐1R signalling pathway through control of IGFBPs IGFBP‐3 mRNA and secreted IGFBP‐6). In addition, IGFBP‐3 and IGFBP‐6 secretion may be a part of defence mechanism, and secreted IGFBPs prolonged half‐life of IGF‐1 and IGF‐2, against blocking IGF‐1 and IGF‐2 in AGS cells. Moreover, downstream pathway inhibition was related with activation of Akt (S473).

To investigate the expression levels of intracellular EMT‐related molecules, which are associated with the ability for invasion, we examined the protein expression of E‐cadherin, Snail and vimentin. Despite the very low E‐cadherin levels observed, no significant differences were detected between the control and BI836845‐treated groups. Snail, which inhibits the activation of E‐cadherin, showed higher expression levels in AGS than in AGS‐EBV cells. In addition, neither AGS nor AGS‐EBV treatment groups showed any changes in Snail levels after treatment. Compared to both control groups, vimentin expression levels in AGS‐EBV were reduced after BI836845 treatment, whereas those in AGS remained unchanged (Figure 3D).

Vimentin expression was down‐regulated after BI836845 treatment in AGS‐EBV but not in AGS cells. We hereby conjectured that the ability to invade may be regulated by vimentin expression in AGS‐EBV cells independently of E‐cadherin and Snail.

### 3.4 Apoptosis or cell cycle arrest was not observed after BI836845 treatment in AGS‐EBV

**Figure 4:**
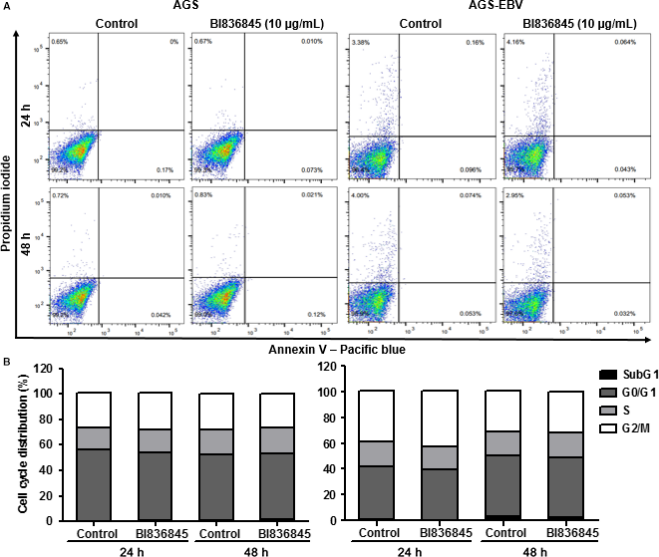
Apoptosis and cell cycle analyses during BI836845 treatment of AGS and AGS‐EBV cells. (A) Apoptotic cell death was measured at 24 h and 48 h using Annexin V/propidium iodide (PI) double staining and FACS. (B) Cell cycle analysis of unsynchronized AGS and AGS‐EBV cells was conducted during 24 h and 48 h with PI staining using FACS. Both AGS and AGS‐EBV cells were treated with 10 μg/mL BI836845

We performed Annexin V/PI double‐staining assay to determine whether the reduction in cell viability was induced by apoptosis. As shown in Figure 4A, the proportion of cell death in AGS‐EBV cells was approximately 4 times greater than that in AGS cells. Neither AGS nor AGS‐EBV cells showed any increment in apoptosis after treatment with 10 μg/mL BI836845.

When we performed cell cycle analysis using PI staining, no significant cell cycle arrest was observed in AGS and AGS‐EBV cells after BI836845 treatment (Figure 4B). Baseline sub‐G1 proportion was approximately 1.0% for both cell lines (AGS and AGS‐EBV). Specifically for AGS‐EBV, both control and BI836845 treated group increased to a 3.0% sub‐G1 proportion at 48 hours.

These results indicate that the reduction in proliferation in AGS‐EBV compare to AGS may be influenced by the increase in natural cell death. However, inhibition of proliferation and cell viability in BI836845‐treated AGS‐EBV cells resulted neither from apoptotic cell death nor from cell cycle arrest.

### 3.5 BI836845 inhibits progression of AGS‐EBV cells into S phase

**Figure 5:**
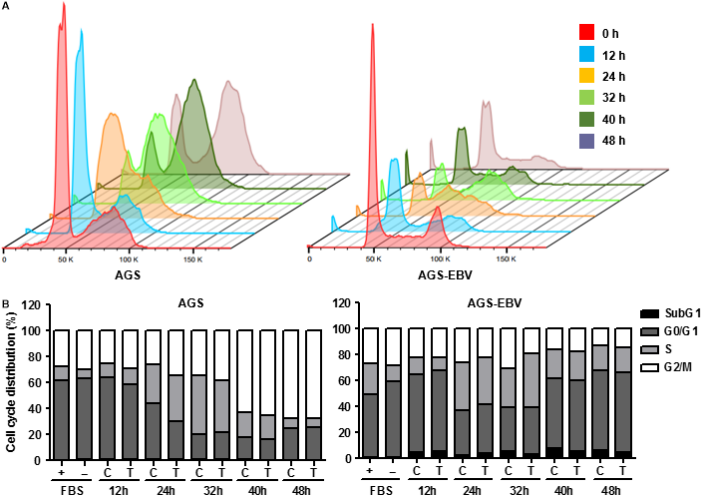
AGS and AGS‐EBV cells were synchronized to G0/G1 to evaluate cell cycle delay. (A) To compare changes in cell cycle progression, synchronized AGS and AGS‐EBV cells are shown in 3D charts. (B) Cell cycle progression was evaluated using propidium iodide staining of synchronized AGS and AGS‐EBV cells. Both AGS and AGS‐EBV cells were harvested after 48 h of synchronization at 0, 12, 24, 32, 40 and 48 h. + (FBS), unsynchronized control cells at 0 h; ‐ (FBS), synchronized cells without BI836845 treatment at 0 h; C, Control without BI836845 treatment; T, treatment with 10 μg/mL BI836845

To further investigate the possible mechanism of proliferation inhibition, we performed cell cycle analysis with G0/G1 synchronization using serum starvation, because no cell cycle arrest could be observed in normal culture conditions. After synchronization, the G0/G1 proportion in AGS and AGS‐EBV cells was 62.0% and 58.2%, respectively. Interestingly, cell cycle progression of AGS‐EBV cells was faster than that of AGS cells (Figure 5A).

After 24 hours of G0/G1 phase synchronization, the transition from G0/G1 to S phase increased from 30.2% to 34.7% during BI836845 treatment in AGS cells. No other significant differences in cell cycle progression were subsequently observed between control and BI836845‐treated groups. In contrast, cell cycle progression from G0/G1 to S phase after 32 hours was delayed, increasing from 29.9% to 41.3% during BI836845 treatment in AGS‐EBV cells. This delay in cell cycle progression was continuously maintained for 40 and 48 hours in the treated AGS‐EBV cells (Figure 5B).

These results indicate that the inhibition of cell proliferation and reduction in cell viability in treated AGS‐EBV cells may result from a delayed cell cycle progression into S phase.

## 4 DISCUSSION

To optimize genome replication, most viruses manipulate the host cell environment and the cell cycle. Many of the EBV‐specific viral factors were identified due to their functional impact on various cell types.^22^, ^23^ Most of these studies, however, limited their focus to viral gene expression and their function. In our study, we extend our focus to the biological changes in gastric cancer cells after EBV infection and also on possible target signalling pathways.

The EBV‐infected gastric cancer cell line (NUGC‐3) and EBV‐positive NPC cell line showed increased IGF‐1 mRNA and secreted IGF‐1 levels compared to parental cell lines, which suggest that IGF‐1 mediates cell proliferation.^17^, ^24^ In our results, IGF‐1 mRNA levels did not increase, although secreted IGF‐1 levels increased in AGS‐EBV cells (Figure 1C). As well known, secreted IGF‐1 stimulates IGF‐1R phosphorylation. Although the mRNA and protein expression levels of most genes we examined were down‐regulated in AGS‐EBV cells, phospho‐IGF‐1R levels were increased in AGS‐EBV cells. When EBV infects cells, it regulates growth of the host cell and activates selective pathways to increase the efficiency of viral factor synthesis.^25^, ^26^ Our results suggest that EBV may use the IGF‐1R pathway to adapt to the host environment in the AGS cell line.

BI836845 treatment of AGS and AGS‐EBV cells resulted in reduced levels of phospho‐IGF‐1R and phospho‐AKT, with greater reduction in AGS‐EBV cells. Secreted IGFBP‐3 and IGFBP‐6 levels were particularly increased in the AGS treatment group. Circulating IGFBPs are known to increase IGF‐1 and IGF‐2 stability and transport in the tissue, and IGFBP‐3 and IGFBP‐6 stabilize IGF‐1 and IGF‐2, respectively.^27^ In our experiments, resistance to BI83685 in AGS cells was observed in concordance with increased IGFBP‐3 and IGFBP‐6 secretion.

Levels of mRNA or cytosol IGFBPs are associated with tumour aggressiveness, and the underlying mechanisms are complex and include both IGF‐dependent and IGF‐independent pathways.^28^ In our results, we observed that IGFBP‐3 mRNA levels were greatly increased in AGS‐EBV cells (Figure 1A). IGFBP‐3 is known to have various binding partners and is associated with different cellular functions.^29^ Several studies have reported an association of elevated mRNA expression levels of IGFBP‐3 with cell growth inhibition, namely in breast and prostate cancer cell lines. These mechanisms are mediated by IGF‐independent pathways.^30^, ^31^, ^32^ In our result, IGFBP‐3 mRNA expression was reduced only in the AGS‐EBV treatment group. IGF‐independent cell death by IGFBP‐3 mRNA regulation may be increased by EBV infection, which in turn increases IGF dependence and BI836845 sensitivity on AGS‐EBV treatment group.

We also observed that while cell cycle progression was faster in AGS‐EBV cells compared to that of AGS cells, cell proliferation was in fact slower in AGS‐EBV (Figures 2A and 4A). The number of necrotic AGS‐EBV cells was increased by fourfold compared to AGS cells. Abnormal regulation by the virus or increment of nuclear IGFBP‐3 level (Supplementary 2) may lead to natural cell death, and this regulation conjectured to cause slower proliferation rate of AGS‐EBV. Although the experiment was tested on different type of cancer (bone osteosarcoma), accumulation of nuclear IGFBP‐3 induced apoptosis with proteolysis.^33^ In our results, inhibition of proliferation did not occur as a result of apoptosis or cell death in AGS‐EBV cells treated with BI836845 (Figure 2B and 4A‐B), but from a delay in entering the S phase of the cell cycle (Figure 5B). One study reported similar blocking of IGF‐1R signalling activation that induced cytostatic effects in colorectal cancer cells. Although the cancer cell line model is different from our study, blocking the IGF signalling pathway using tyrosine kinase inhibitors may have effects similar to those described in this work.^34^

In contrast to the increased baseline invasiveness of AGS‐EBV cells compared with AGS cells, (Figure 2C‐D), expression of EMT markers was reduced in AGS‐EBV cells (Figure 3D). EBV infection of AGS cells led to suppression of most baseline protein expression, a common characteristic of virus‐infected cells. Particularly, expression of EMT‐related molecules has been shown to be regulated by EBV‐specific molecules.^35^ In our result, mRNA levels of IGFBP‐6 in AGS cells increased when treated with BI836845 (Figure 3A), and AGS cells treated with 10 μg/mL of BI836845 showed 3 times higher ability for invasion than the control group (Figure 2D). In contrast, mRNA level of IGFBP‐6 in AGS‐EBV was decreased after BI836845 treatment. Interestingly, high mRNA level of IGFBP‐6 promotes cancer migration and invasion in an IGF‐independent manner.^36^

It has been shown that IGF‐1‐dependent secretion of IGFBP‐3 induces angiogenesis and positively regulates the expression of pro‐angiogenic molecules.^37^ In our results, IGFBP‐3 secretion was only observed in treated AGS cells and their invasion to the microenvironment was increased compared to AGS‐EBV. IGFBP‐3 and IGFBP‐6 have very complex IGF‐dependent and IGF‐independent functions, which remains mostly unclear. EBV‐infected AGS cells may regulate their growth and invasiveness using IGFBP‐3 and IGFBP‐6 inside and outside cells. Inhibition of vimentin expression, independent of other EMT proteins Snail and E‐cadherin, was observed in the AGS‐EBV treatment group (Figure 3D). This suggests that vimentin might play an independent role in the regulation of AGS‐EBV cell invasiveness.

As mechanistic studies of IGFBP‐3 and IGFBP‐6 were not accomplished in our study, future studies will be needed to evaluate the intra‐ and extracellular functional activity.

## 5 CONCLUSIONS

In our study, we demonstrated that the IGF‐1R‐related pathway dependency of both proliferation and invasion was changed by EBV infection in a gastric cancer cell line. Moreover, EBV‐infected cancer cells became sensitive to BI836845. Also we suggested that regulation of IGFBP‐3 and IGFBP‐6 had important roles on proliferation and invasion of EBVaGC. Our results suggest that although limited in scope due to validation in a single EBV‐infected gastric cell line, IGF‐1R pathway inhibition might be an effective therapeutic target in EBVaGC.

## CONFLICT OF INTEREST

The authors confirm that there are no conflicts of interest.
