# Supplementary figures and images for: Regulation of proliferation and invasion by the IGF signalling pathway in Epstein‐Barr virus‐positive gastric cancer
Source: J Cell Mol Med. 2018 Sep 24;22(12):5899–908. doi: 10.1111/jcmm.13859 (PMC6237558; doi:10.1111/jcmm.13859)

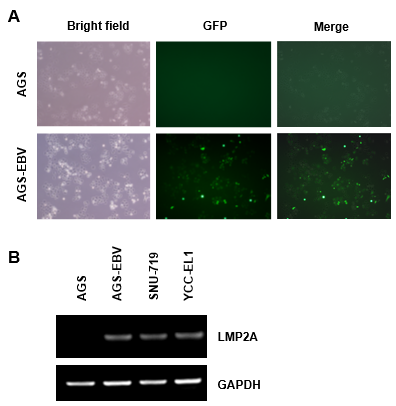

Supplement: Supplementary file 1 [file JCMM-22-5899-s001.tif]

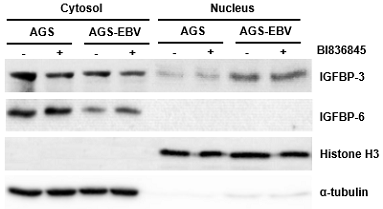

Supplement: Supplementary file 2 [file JCMM-22-5899-s002.tif]
